# Thermostability and excision activity of polymorphic forms of hOGG1

**DOI:** 10.1186/s13104-019-4111-9

**Published:** 2019-02-18

**Authors:** Kathryn D. Mouzakis, Tiffany Wu, Karl A. Haushalter

**Affiliations:** 1 Present Address: Department of Chemistry and Biochemistry, Loyola Marymount University, 1 LMU Drive, LSB #284, Los Angeles, CA 90045 USA; 2 Present Address: Vascular & Interventional Specialists of Orange County, 1140 W. La Veta Avenue, Suite 850, Orange, CA 92868 USA; 3 Departments of Chemistry and Biology, Harvey Mudd College, 301 Platt Blvd., Claremont, CA 91711-5990 USA

**Keywords:** DNA repair, 8-Oxoguanine, DNA glycosylase, Mutation, Cancer

## Abstract

**Objectives:**

Reactive oxygen species (ROS) oxidize guanine residues in DNA to form 7,8-dihydro-oxo-2′-deoxyguanosine (8oxoG) lesions in the genome. Human 8-oxoguanine glycosylase-1 (hOGG1) recognizes and excises this highly mutagenic species when it is base-paired opposite a cytosine. We sought to characterize biochemically several hOGG1 variants that have been found in cancer tissues and cell lines, reasoning that if these variants have reduced repair capabilities, they could lead to an increased chance of mutagenesis and carcinogenesis.

**Results:**

We have over-expressed and purified the R46Q, A85S, R154H, and S232T hOGG1 variants and have investigated their repair efficiency and thermostability. The hOGG1 variants showed only minor perturbations in the kinetics of 8oxoG excision relative to wild-type hOGG1. Thermal denaturation monitored by circular dichroism revealed that R46Q hOGG1 had a significantly lower T_m_ (36.6 °C) compared to the other hOGG1 variants (40.9 °C to 43.2 °C). Prolonged pre-incubation at 37 °C prior to the glycosylase assay dramatically reduces the excision activity of R46Q hOGG1, has a modest effect on wild-type hOGG1, and a negligible effect on A85S, R154H, and S232T hOGG1. The observed thermolability of hOGG1 variants was mostly alleviated by co-incubation with stoichiometric amounts of competitor DNA.

**Electronic supplementary material:**

The online version of this article (10.1186/s13104-019-4111-9) contains supplementary material, which is available to authorized users.

## Introduction

Reactive oxygen species oxidize the DNA base guanine, forming mutagenic 7,8-dihydro-oxo-2′-deoxyguanosine (8oxoG) [1, 2]. Mispairing of 8oxoG with adenine during DNA replication results in G-to-T mutations. In humans, 8oxoG is targeted by the base excision repair pathway [3, 4], which is initiated when 8-oxoguanine DNA glycosylase-1 (hOGG1) catalyzes the hydrolysis of the *N*-glycosidic bond linking 8oxoG and deoxyribose in DNA [5–10].

Given the role of hOGG1 in preventing mutagenesis, a connection between deficiencies in hOGG1 activity and cancer seems plausible, but when the evidence for such a connection is examined, the data presents a mixed picture (reviewed in [9, 11–15]). Therefore, additional functional information about the naturally occurring hOGG1 variants would be beneficial for this analysis.

In this study, we investigated the repair efficiency and protein stability of four variants of hOGG1: R46Q, A85S, R154H, and S232T. The R46Q hOGG1 variant was first discovered in a human lung cancer cell line [16] and has reduced repair activity compared to wild-type hOGG1 [17, 18]. The R154H hOGG1 variant arises from somatic mutation and was first identified in a gastric cancer cell line [19]. In addition to having a lower activity with its native substrate (8oxoG base-paired with cytosine), R154H hOGG1 also displays decreased specificity for the base opposite 8oxoG [17, 20]. Molecular dynamics simulations show that both the R46Q hOGG1 variant and the R154H hOGG1 variant feature a reorganized and slightly wider active site compared to wild-type hOGG1 [21]. Less is known about the two final variants studied here: A85S hOGG1, first identified in a lung cancer patient [22]; and S232T hOGG1, first identified in a human kidney tumor [22]. Both of these variants were shown to be capable of excising 8oxoG [18], but this does not exclude the possibility of a more subtle defect in repair kinetics or stability. All four hOGG1 variants in this study were overexpressed in bacteria, purified, and then studied biochemically.

## Main text

### Methods

#### Generating hOGG1 proteins

For detailed experimental methods, see Additional file 1. Briefly, the full-length wild-type α-hOGG1 coding sequence was subcloned into pET-28a (Novagen, Madison, WI) to synthesize a hOGG1/pET-28a construct that produces a fusion protein with an N-terminal hexahistidine tag. Site-directed mutagenesis using the QuikChange Site-Directed Mutagenesis Kit (Stratagene, La Jolla, CA) generated the over-expression plasmids for the hOGG1 variants R46Q, A85S, R154H, and S232T. Bacteria cells transformed with the appropriate plasmid and induced to express protein were harvested by centrifugation and lysed. The resulting protein extract was subjected to a two-column purification protocol to yield purified hOGG1 protein (for gel analysis of purified proteins, see Additional file 2). The remaining N-terminal hexahistidine tag has been shown to have negligible effect on the DNA glycosylase activity of hOGG1 [23].

#### Preparation of DNA substrates

Oligonucleotides were purchased from Operon Biotechnologies (Huntsville, AL). For fluorescently labeled substrates, the Cy5 label was incorporated during DNA synthesis and for radiolabeled substrates, the 5′ end of the 8oxoG containing strand was radiolabeled with γ-^32^P-ATP using T4 polynucleotide kinase. Duplexes were formed by annealing to the complementary strand. Prior to use, radiolabeled DNA substrates were mixed in a 1/10 ratio with identical, unlabeled DNA duplexes. The sequences of the DNA substrates are listed below:

**8oxoG/C**

5’-ATCAGTGAG[8oxoG]CAGTCATCAG-3’

3’-TAGTCACTC C GTCAGTAGTC-5’

**Cy5-8oxoG/C**

$$ \begin{aligned} &\texttt{5}'\texttt{-}[\texttt{Cy5}]{\texttt{ATCAGTGAG}}[\texttt{8oxoG}]\texttt{CAGTCATCAG-3}' \\ & \qquad\;\texttt{3}'\texttt{-TAGTCACTC}   \quad \; \texttt{C}   \quad \;\, \texttt{GTCAGTAGTC-5}' \end{aligned}$$

**G/C**

5’-ATCAGTGAGGCAGTCATCAG-3’

3’-TAGTCACTCCGTCAGTAGTC-5’

#### DNA glycosylase excision assays

The DNA glycosylase excision assay was adapted from the single- and multiple turnover assays described by David et al. [23] (for more experimental details, see Additional file 1). Briefly, the radiolabeled 8oxoG/C substrate was incubated with hOGG1 protein at 37 °C and, at specified time points, aliquots were removed and quenched. Reaction products were resolved by denaturing polyacrylamide electrophoresis. All time courses were replicated at least three times. For the thermolability studies, the standard hOGG1 cleavage assay was modified by pre-incubating hOGG1 for 90 min at either 4 °C or 37 °C prior to the reaction. In a variation of this experiment, hOGG1 was co-incubated with undamaged DNA (G/C DNA duplex above) during this 90-min step. The products were analyzed as described above for the standard assay, except that the DNA substrate employed was the Cy5-8oxoG/C duplex.

#### CD spectroscopy

Circular dichroism (CD) spectra of wild-type hOGG1 and hOGG1 variants were obtained with a Jasco-715 spectropolarimeter. Data were recorded as the temperature was increased from 10.0 °C to 95.0 °C at a rate of 1 °C min^−1^. All denaturations were performed in triplicate. The Jasco-715 software was used to smooth the data and calculate the melting points based on the change in the molar ellipticity [θ] (degree cm^2^ dmol^−1^) at 222 nm with rising temperature.

### Results

#### Kinetic analysis of glycosylase activity

**Fig. 1:**
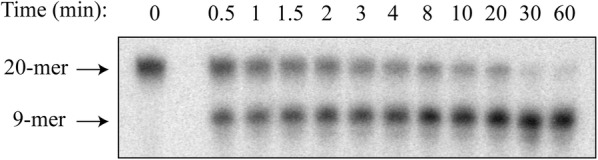
Representative time course of hOGG1 activity. One strand of this 20 bp duplex contains 8oxoG at the 10th position from the radiolabeled 5′ end. The substrate was incubated with hOGG1, in this case wild-type, for the time indicated, quenched with hot alkali, and then analyzed by denaturing polyacrylamide gel electrophoresis. The 20-mer band corresponds to non-cleaved DNA, while the 9-mer band corresponds to DNA processed by hOGG1

To measure activity of the hOGG1 variants, we utilized a standard DNA glycosylase activity assay in which labeled, double-stranded DNA containing a single 8oxoG opposite cytosine was treated with DNA glycosylase for varying amounts of time and then quenched. A typical time course of product formation from the enzyme reaction, as resolved by gel electrophoresis, is shown in Fig. 1. For each hOGG1 variant, the amount of product formed at each time point was quantified and averaged over a minimum of three replicates, as plotted in Additional file 3. The time course of product (P) formation was fit to Eq. 1, in which a rapid burst phase with amplitude *A*_*0*_ and rate constant *k*_*1*_ is followed by a slower, linear phase with rate constant *k*_2_ [24].

1 $$ [P] = A_{o} \left( {1 - e^{{ - k_{1} t}} } \right) + k_{2} t $$

The introduced mutations in hOGG1 had a modest effect on the kinetics of the burst phase of the reaction, as judged by the values for the rate constant *k*_*1*_ (Table 1).

**Table 1:** Summary of DNA glycosylase activity rate constants for hOGG1 variants

hOGG1 variant	*A*_*0*_ (nM)	*k*_*1*_ (min^−1^)	*k*_*2*_ (nM min^−1^)
Wild-type	14.2 ± 0.3	1.4 ± 0.1	0.09 ± 0.01
R46Q	10.5 ± 0.6	2.0 ± 0.6	0.14 ± 0.03
A85S	14.3 ± 0.3	1.6 ± 0.1	0.08 ± 0.01
R154H	8.4 ± 0.6	0.23 ± 0.03	0.14 ± 0.01
S232T	10.5 ± 0.5	0.8 ± 0.1	0.15 ± 0.02

Compared to wild-type hOGG1 (*k*_*1*_ = 1.4 ± 0.1 min^−1^), the most severely affected variants for the burst phase were R154H hOGG1 (*k*_*1*_ = 0.23 ± 0.03 min^−1^) and S232T hOGG1 (*k*_*1*_ = 0.8 ± 0.1 min^−1^). The slower linear phase of the reaction was slightly faster for most hOGG1 variants relative to wild-type as judged by the rate constant *k*_*2*_, which may reflect reduced affinity for the product of the reaction.

#### Thermostability

To see how the mutations in hOGG1 affect protein folding, thermal denaturation experiments monitored by CD spectroscopy was performed for each hOGG1 variant (for unfolding curves see Additional file 4). The resulting data were analyzed to determine the melting temperature (T_*m*_). The R46Q substitution significantly destabilizes hOGG1 relative to wild-type (T_*m*_ = 36.6 ± 0.5 °C compared to T_*m*_ = 41.8 ± 0.3 °C). In contrast, the A85S and S232T hOGG1 variants (both T_*m*_ = 42.2 °C ± 0.1) have a similar thermostability compared to wild-type hOGG1. Finally, the R154H hOGG1 variant is slightly stabilized (T_*m*_ = 43.2 ± 0.3 °C).

**Fig. 2:**
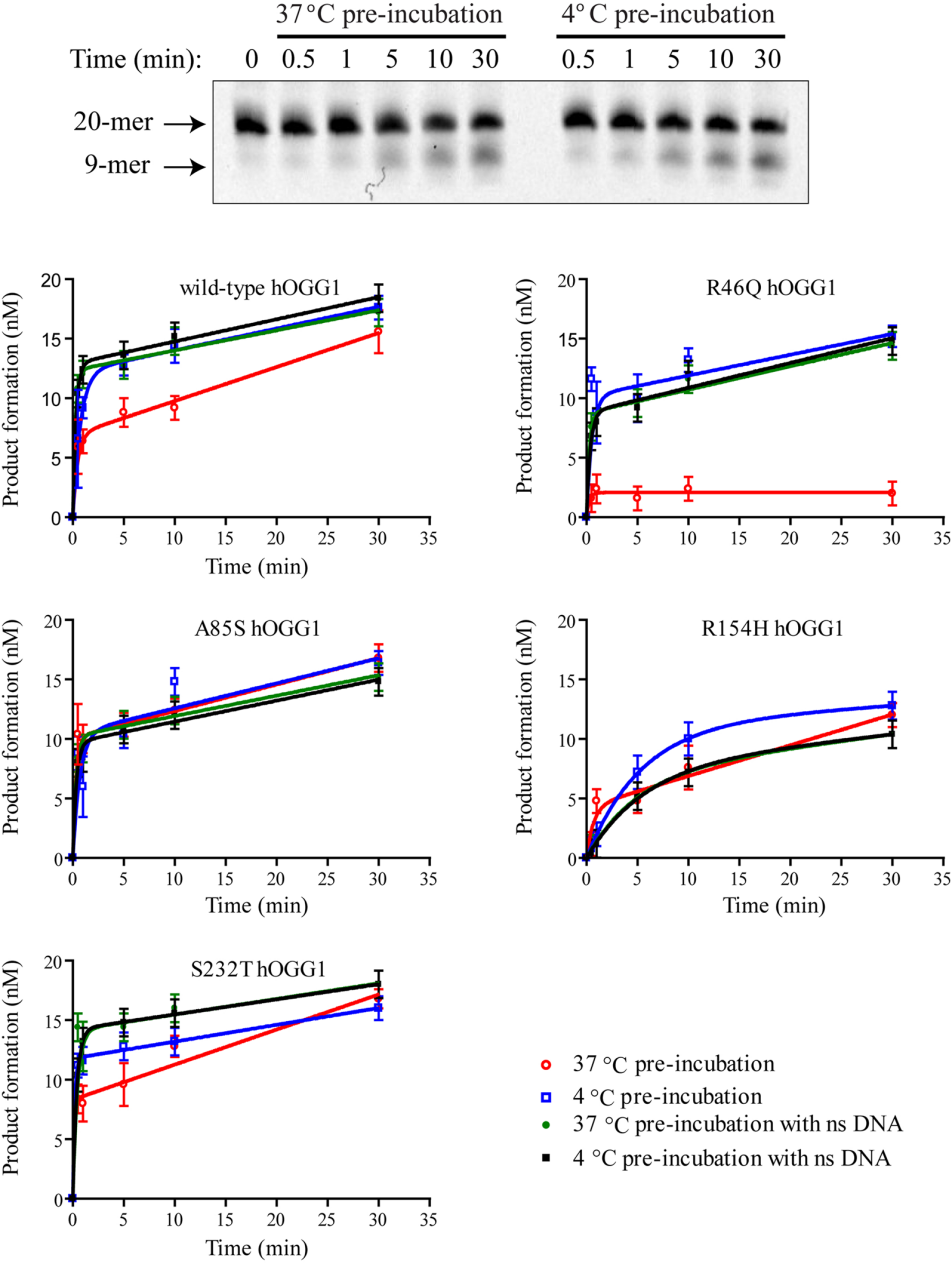
Thermolability of excision activity for hOGG1 variants. The hOGG1 variants’ enzyme activities were compared with or without thermal challenge. The top panel shows a representative gel image, in this case for the R46Q hOGG1 variant in the presence of non-specific DNA. In the lower panels, product formation, as measured from the glycosylase activity assay, is plotted as a function of time for each hOGG1 variant. Prior to the excision assay, hOGG1 variants were pre-incubated at either 37 °C (circles) or 4 °C (squares). The pre-incubation was carried out in the absence (open markers) or the presence (filled markers) of stoichiometric undamaged DNA. For each hOGG1 variant the glycosylase assay was replicated a minimum of three times at each condition. Error bars represent the standard deviation

To investigate how the observed differences in hOGG1 T_*m*_ affects excision activity, a thermolability study was undertaken. Prior to the excision assay, each hOGG1 variant was pre-incubated at either 4 °C or 37 °C for 90 min. The hOGG1 activity was then assessed with the glycosylase activity assay described above. Figure 2 shows a representative gel image (top panel) and the quantified results for each variant (bottom panels). As expected from the thermal denaturation results, the R46Q hOGG1 excision activity was reduced to near background levels following an extended pre-incubation at 37 °C (Fig. 2—compare red open circles and blue open squares). The other hOGG1 variants showed more mild reductions or no reduction in excision activity after the 37 °C pre-incubation.

For the MutY DNA glycosylase, incubation with undamaged DNA was previously observed to be protective for enzyme activity [24]. To see if this effect similarly impacts the hOGG1 variants, the thermolability assays were repeated in the presence of stoichiometric competitor DNA lacking 8oxoG (Fig. 2—green filled circles and black filled squares). Significantly, for all variants, regardless of T_*m*_, the differences in activity following the 4 °C and 37 °C pre-incubations were nearly abolished by the addition of undamaged DNA. For the thermolabile R46Q hOGG1 variant, the undamaged DNA provided almost complete protection from thermal denaturation during the 37 °C pre-incubation.

### Discussion

Translating knowledge of variations in DNA repair genes into useful information about cancer susceptibility is a complex problem [25]. In one mathematical model for base excision repair, the steady state prevalence of mutagenic lesions is insensitive to mild variations in the catalytic activity of the DNA glycosylase [26]. According to this model, a 50% reduction in the turnover number of hOGG1 is predicted to lead to only a 3% rise in the steady-state level of DNA damage [26]. Using this model to aid interpretation of our kinetic results, we predict that three of the mutations in hOGG1 studied here (R46Q, A85S, S232T) are unlikely to yield significantly elevated mutagenesis rates due to slower repair of 8oxoG. The hOGG1 variant that could yield significantly elevated rates of mutagenesis is the R154H variant, which retains only ~ 16% of wild-type activity. Furthermore, R154H hOGG1 has been shown previously to have relaxed specificity for the base opposite 8oxoG, which further drives mutagenesis [17, 20].

Additionally, this report shows that the R46Q hOGG1 variant is thermolabile by both CD thermal denaturation and an activity assay. The high-resolution structure of hOGG1 bound to DNA reveals that R46 serves as a stabilizing scaffold to connect three secondary structure elements: αE, βG, and the loop between αA and βB (see Additional file 5 and Ref. [20]). Introducing the R46Q mutation would most likely disrupt the hydrogen bonds that stabilize the secondary structure junction in this region of the protein. The sensitivity of R46Q hOGG1 to thermal denaturation is likely the reason that this variant has been previously reported to have reduced activity [17, 18]. On the other hand, we observed a dramatic reduction in thermolability upon co-incubation with competitor DNA lacking 8oxoG. For hOGG1 variants destabilized by mutations in structurally important residues, such as R46, the added stability gained upon DNA binding is apparently sufficient to stabilize the folded and active conformation of the enzyme [27, 28].

Both R46 and R154 are completely conserved in OGG1 sequences from divergent species (for a multiple sequence alignment see Additional file 6), which is consistent with the detrimental effects of introducing mutations at these positions (this study and References [17, 18, 20]). In contrast, A85 and S232 are not strongly conserved and it is not surprising that proteins with mutations at these positions show no significant difference and only minor differences, respectfully, in activity.

### Conclusions

In this study, the R46Q, A85S, R154H, and S232T hOGG1 variants were characterized biochemically in comparison to wild-type hOGG1. The kinetics of 8oxoG excision by the hOGG1 variants were only mildly changed, with R154H hOGG1 having the greatest loss of activity. In addition, one of the variants, R46Q, showed increased thermolability. Binding to undamaged DNA was protective for all hOGG1 variants, including the thermolabile R46Q. Considering these results, carrying one of these variants of hOGG1 is probably not sufficient by itself to significantly increase the risk of carcinogenesis.

## Limitations

The experiments performed here were conducted in vitro with purified proteins and small oligonucleotide substrates, in contrast to the more complex environment of a living cell. The hOGG1 protein is one component of an interdependent process and variant forms of hOGG1 could potentially increase the likelihood of carcinogenesis when combined with other genetic and environmental risk factors.

## Additional files

**Additional file 1.** Detailed experimental methods.

**Additional file 2.** SDS-PAGE analysis of purified wild-type hOGG1 and hOGG1 variants. Proteins were over-expressed in bacteria and purified by two chromatography steps. Analysis was performed with a 12% SDS polyacrylamide gel, stained with Coomassie Blue. MW = Kaleidoscope molecular weight ladder (Bio-Rad), WT = wild-type.

**Additional file 3.** Time course of DNA glycosylase activity for the different hOGG1 variants. In each case, DNA substrate (20 nM) was incubated with hOGG1 (100 nM) for varying times prior to the reaction being quenched with sodium hydroxide. The double-stranded 20-mer DNA substrates contained a centrally located single 8oxoG base opposite cytosine. The products of the DNA glycosylase reaction were separated by denaturing polyacrylamide gel electrophoresis and the band intensities quantified. For each hOGG1 variant the glycosylase assay was replicated a minimum of three times. Error bars at the individual time points represent the standard deviation. The resulting data was averaged and fit to Eq. 1. The large variance in product formation for the R46Q hOGG1 variants was observed over numerous replicates of the glycosylase assay.

**Additional file 4.** Thermal denaturation of hOGG1 variants. Circular dichroism (CD) spectra were recorded for each protein sample at a concentration of 0.20 mg mL^−1^. The molar ellipticity [θ] (degree cm^2^ dmol^−1^) at 222 nm was recorded as the temperature was increased from 10.0 to 95.0 °C at a rate of 1 °C min^−1^ and the resulting data was normalized to provide the fraction denatured. The average values from three replicate denaturations are plotted.

**Additional file 5.** Structural analysis of the amino acid residues mutated in hOGG1 variants. Analysis based on the original published structure of hOGG1 bound to DNA [20].

**Additional file 6.** Multiple sequence alignment for OGG1 from diverse organisms. Yellow bars highlight amino acid residues that were varied in this study (R46, A85, R154, and S232). Residues that participate directly in catalysis (K249 and D268) are marked in red. The secondary structure annotation is based on the high-resolution crystal structure of K249Q hOGG1 bound to DNA [20]. The conserved HhH-GPD motif is highlighted in purple. Sequences were aligned using ClustalW2 [29]. The Genbank accession numbers for the sequences used are as follows: *Homo sapiens*, [GenBank:AAB61340.1]; *Macaca mulatta*, [GenBank:XP_001096322.1]; *Bos taurus*, [GenBank:NP_001073754.2]; *Mus musculus*, [GenBank:NP_035087.3]; *Rattus norvegicus*, [GenBank:NP_110497.1]; *Arabidopsis thaliana*, [GenBank:CAC83625.1]; *Drosophila melanogaster*, [GenBank:NP_572499.2].

## Abbreviations

8oxoG: 7,8-dihydro-oxo-2′-deoxyguanosine
CD: circular dichroism
hOGG1: human 8-oxoguanine glycosylase 1
