# Supplementary material for: Thermostability and excision activity of polymorphic forms of hOGG1
Source: BMC Res Notes. 2019 Feb 18;12:92. doi: 10.1186/s13104-019-4111-9 (PMC6379936; doi:10.1186/s13104-019-4111-9)
Supplement: Supplementary file 1 — Additional file 1. Detailed experimental methods. [file 13104_2019_4111_MOESM1_ESM.pdf]

Supplementary Detailed Methods

Production and purification of hOGG1 proteins

A plasmid containing the full-length wild-type α-hOGG1 coding sequence was generously provided by Gregory L. Verdine and co-workers [5]. The complete hOGG1 coding sequence was PCR amplified with primers appended with BamHI and XhoI restriction sites and the resulting PCR product was subcloned into pET-28a (Novagen, Madison, WI) at the BamHI and XhoI restriction sites. DNA sequencing confirmed successful subcloning and that no mutations were introduced. The final hOGG1/pET-28a construct produces a fusion protein with an N-terminal hexahistidine tag, which does not interfere with hOGG1's excision activity when the fusion protein is used [24]. Site-directed mutagenesis of the hOGG1/pET-28a construct was accomplished using the QuikChange Site-Directed Mutagenesis Kit (Stratagene, La Jolla, CA) to create over-expression plasmids for the hOGG1 variants R46Q, A85S, R154H, and S232T. DNA sequencing verified that the hOGG1 variant constructs contained no secondary mutations.

To produce the hOGG1 protein, the over-expression construct was transformed into BL21(DE3) cells (Novagen, Madison, WI) and a one liter culture was grown at 37 °C with continuous shaking in LB supplemented with kanamycin. Expression was induced in mid-log phase (OD = 0.5-0.7) by the addition of isopropyl-b-D thiogalactopyranoside (IPTG) to a final concentration of 400-1000 µM. The culture was then incubated with continuous shaking at 30 °C for 3 to 4 hours (wild-type, R154H) or 16 °C overnight (R46Q, A85S, S232T). Cells were pelleted by centrifugation for 10 minutes at 4 °C at 8,000 *g* and the cell pellet was resuspended in 40 mL of Buffer A (20 mM Tris-Cl, 50 mM NaCl, 5 mM imidazole, 10% (v/v) glycerol, pH 7.5). A protease inhibitor cocktail (Research Products International, Mt. Prospect, IL) was added to the

suspension and the cells were lysed by passage through a French Press. The lysate was clarified by a 25-minute centrifugation at 4 °C and 24,000 *g*. The clarified lysate was combined with 2.5 mL (bed volume) of Ni-NTA resin (Qiagen, Valencia, CA) that had been pre-washed with Buffer A. The resulting mixture was gently rotated at 4 °C for at least 45 minutes. The clarified lysate-Ni-NTA resin mixture was poured into a gravity column and drained. The resin was washed twice with Buffer A and the hOGG1 protein was eluted with Buffer B (20 mM Tris-Cl, 50 mM NaCl, 500 mM imidazole, 10% (v/v) glycerol, pH 7.5). The eluted protein was dialyzed overnight at 4 °C against Buffer C (20 mM Tris-Cl, 50 mM NaCl, 1 mM EDTA, 10% (v/v) glycerol, pH 7.5). The partially purified protein was loaded onto a Source15S 4.6/100 PE column (GE Healthcare, Piscataway, NJ), washed with Buffer C, and then eluted with a linear gradient from 50 mM to 1000 mM NaCl in Buffer D (20 mM Tris-Cl, 1 mM EDTA, 10% (v/v) glycerol, pH 7.5). Fractions containing significant amounts of pure hOGG1 were pooled and dialyzed against Buffer C overnight at 4 °C. The purified protein was aliquoted into single-use tubes, flash frozen in liquid nitrogen, and stored at -80 °C. Concentrations were determined using the Bradford assay (Bio-Rad, Hercules, CA) with BSA as a standard.

Preparation of DNA substrates

All oligonucleotides were purchased from Operon Biotechnologies (Huntsville, AL). For radiolabeled DNA substrates used in the initial excision kinetics assays, the 5' end of the 8oxoG containing strand was radiolabeled with γ-^{32}P-ATP (Perkin Elmer, Waltham, MA) using T4 polynucleotide kinase (New England Biolabs, Ipswich, MA). Unincorporated radionucleotides were removed by Centri-Spin gel filtration spin columns (Princeton Separations, Adelphia, NJ). For fluorescently labeled DNA substrates used for the thermolability studies, the Cy5 label was incorporated during DNA synthesis and the resulting DNA substrates were stored in the dark. The change from radiolabeled to fluorescently labeled substrates was made for cost and time effectiveness. All duplexes were formed by annealing to the desired complementary strand in

10 mM Tris-Cl (pH 7.0) and 150 mM NaCl. Prior to use, radiolabeled DNA substrates were mixed in a 1/10 ratio with matched, identical DNA duplexes that lacked the radiolabel. The sequences of the DNA substrates are listed below:

```
Cy5-8oxoG/C      5′Cy5-ATC AGT GAG 8oxoG CAG TCA TCA G 3′
                    3′ TAG TCA CTC   C   GTC AGT AGT C 5′

8oxoG/C          5′ ATC AGT GAG 8oxoG CAG TCA TCA G 3′
                 3′ TAG TCA CTC   C   GTC AGT AGT C 5′

G/C              5′ ATC AGT GAG G CAG TCA TCA G 3′
                 3′ TAG TCA CTC C GTC AGT AGT C 5′
```

DNA glycosylase excision assays

The procedure for the DNA glycosylase excision assay was adapted from the single- and multiple turnover assays described by Sheila David and co-workers [24]. Briefly, 20 nM of the radiolabeled 8oxoG/C substrate was equilibrated for 5 minutes at 37 °C in reaction buffer (20 mM Tris-Cl, 10 mM EDTA, 100 μg/mL BSA, pH 7.1). The hOGG1 protein was added to this solution (to a final concentration of 100 nM) and the reaction was incubated at 37 °C. At various time points, 10 μL aliquots were removed from the reaction and were immediately added to 10 μL of stop solution (90% formamide, 1X TBE, 0.04% xylene cyanol, 0.03% bromophenol blue), and 2 μL of 1 M NaOH to quench the reactions. The quenched reaction was then heated at 90 °C for at least 5 minutes. Prior to gel analysis, samples were reheated for an additional 5 minutes at 90 °C and then resolved a 16% denaturing polyacrylamide gel. As shown in Supplementary Figure 2, results were visualized and quantified using the Storm 860 imaging system and ImageQuant TL software (GE Healthcare, Piscataway, NJ). Excision of 8oxoG from DNA by hOGG1 leads to strand scission by two possible pathways: the intrinsic (but slow) β-elimination activity of hOGG1 and hydroxide catalyzed hydrolysis of abasic sites during the post-reaction workup. The fraction of product DNA was calculated by dividing the intensity of the

band corresponding to the 9-mer product by the sum of the intensities of the bands corresponding to the 9-mer product and the 20-mer substrate. Finally, the absolute amount of product formation reported at each time point was calculated by multiplying the input DNA substrate concentration by the fraction of product DNA calculated above. All assays were replicated at least three times.

For the thermolability studies, the standard hOGG1 cleavage assay described above was modified. Prior to the reaction, hOGG1 was diluted to 1 µM in Buffer C and incubated for 90 minutes at either 4 °C or 37 °C. In a variation of this experiment, the diluted hOGG1 was co-incubated with 1 µM of the undamaged G/C DNA duplex (*2.2 Preparation of DNA Substrates*) during this 90-minute step. After pre-incubation, hOGG1 was incubated with DNA and the products were analyzed as described above for the standard assay, except that the DNA substrate employed was the Cy5-8oxoG/C duplex at a final concentration of 40 nM, the undamaged G/C DNA duplex had a final concentration of 100 nM, and that the stop solution lacked the xylene cyanol and bromophenol blue dyes.

CD spectroscopy and thermal denaturation

Circular dichroism (CD) spectra of wild-type hOGG1 and hOGG1 variants were obtained with a Jasco-715 spectropolarimeter with a Peltier-type cell holder and 0.1 cm path length cell. Protein concentrations were 0.20 mg/mL in 20 mM Tris-Cl, 50 mM NaCl, 1 mM EDTA, 10% (v/v) glycerol, pH 7.5. Data were recorded every 0.1 °C as the temperature was increased from 10.0 °C to 95.0 °C at a rate of 1 °C/minute and all denaturations were performed in triplicate. The Jasco-715 software was used to smooth the data and calculate the melting points based on the change in the molar ellipticity [θ] (degree cm^2 $dmol^{-1}$) at 222 nm observed with increasing temperature.
