# Supplementary figures and images for: Thermostability and excision activity of polymorphic forms of hOGG1
Source: BMC Res Notes. 2019 Feb 18;12:92. doi: 10.1186/s13104-019-4111-9 (PMC6379936; doi:10.1186/s13104-019-4111-9)

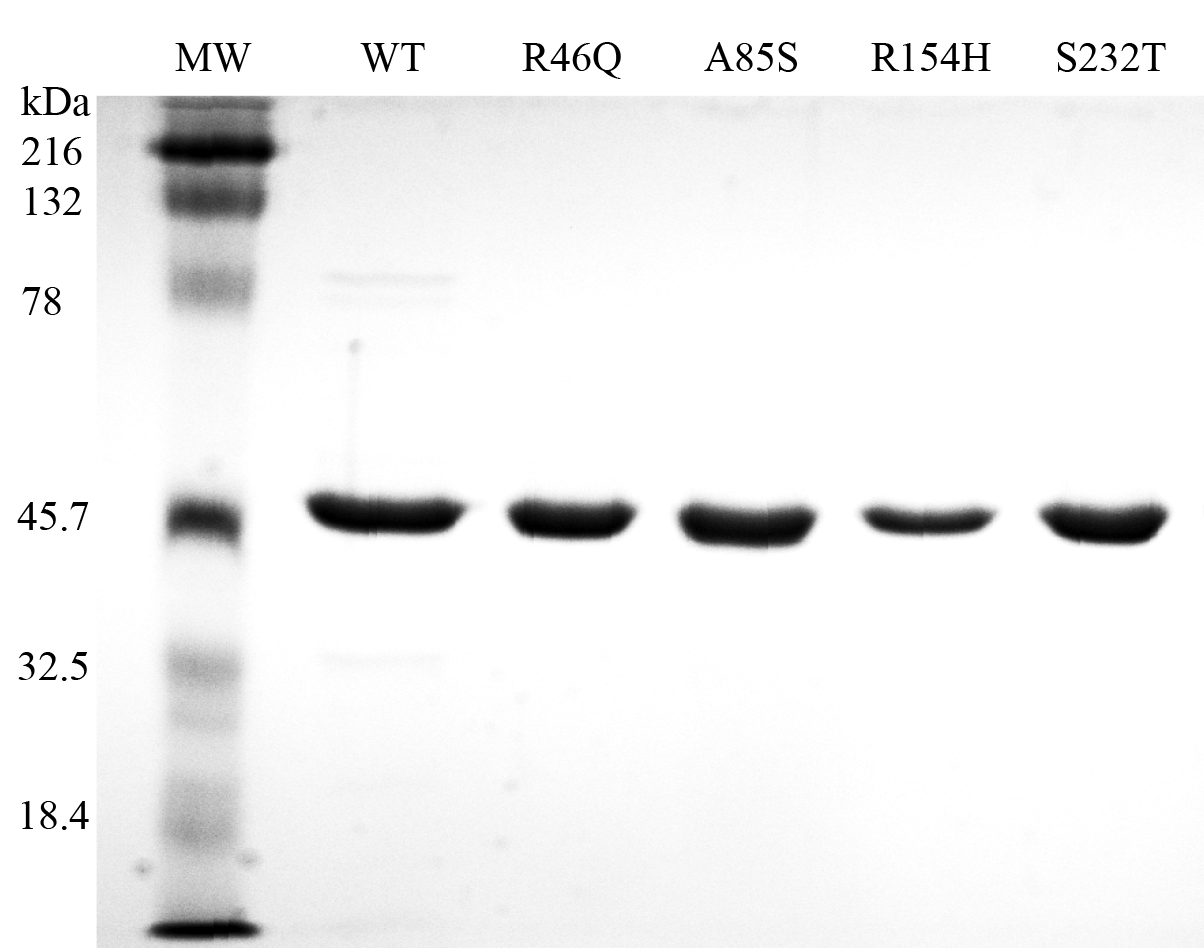

Supplement: Supplementary file 2 — Additional file 2. SDS-PAGE analysis of purified wild-type hOGG1 and hOGG1 variants. Proteins were over-expressed in bacteria and purified by two chromatography steps. Analysis was performed with a 12% SDS polyacrylamide gel, stained with Coomassie Blue. MW = Kaleidoscope molecular weight ladder (Bio-Rad), WT = wild-type. [file 13104_2019_4111_MOESM2_ESM.tif]

(A)

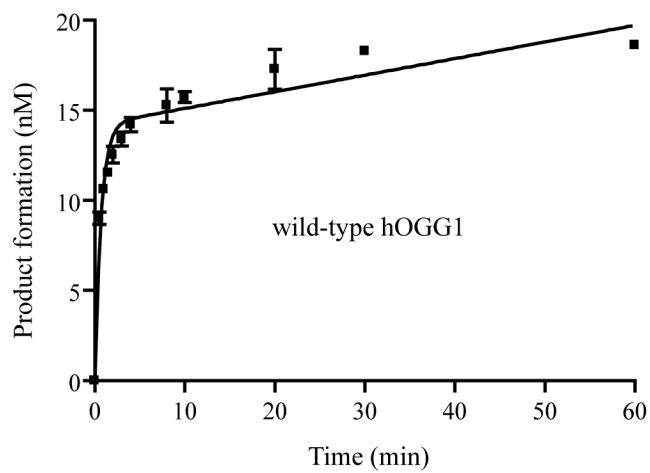

(B)

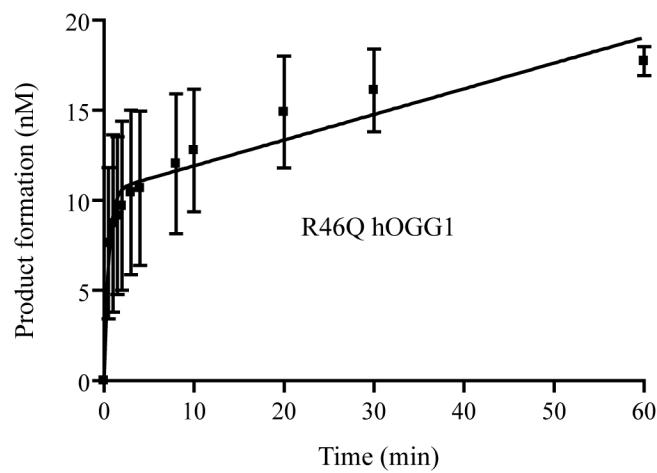

(C)

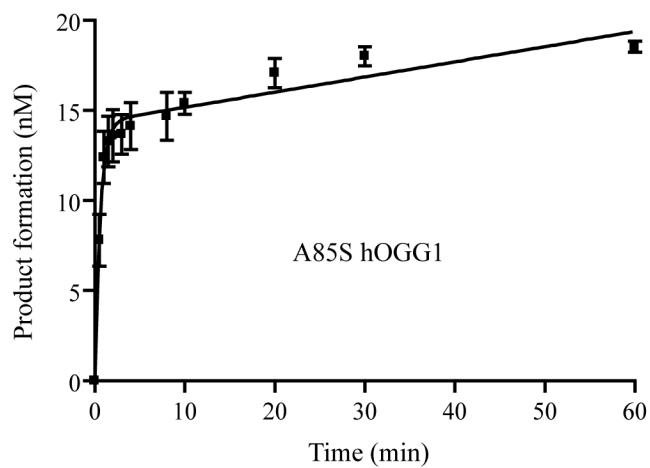

(D)

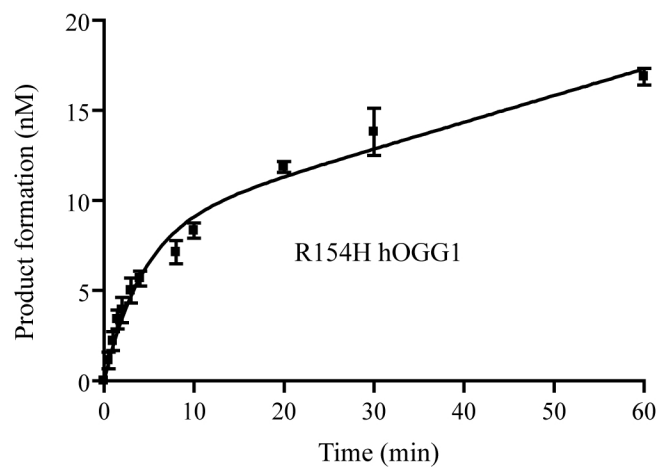

(E)

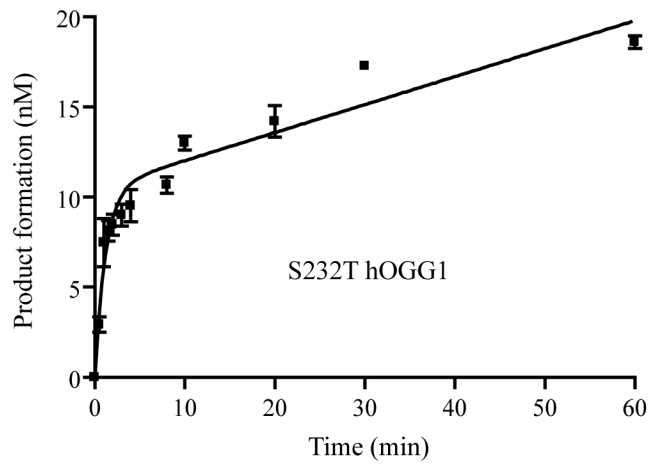

Supplement: Supplementary file 3 — Additional file 3. Time course of DNA glycosylase activity for the different hOGG1 variants. In each case, DNA substrate (20 nM) was incubated with hOGG1 (100 nM) for varying times prior to the reaction being quenched with sodium hydroxide. The double-stranded 20-mer DNA substrates contained a centrally located single 8oxoG base opposite cytosine. The products of the DNA glycosylase reaction were separated by denaturing polyacrylamide gel electrophoresis and the band intensities quantified. For each hOGG1 variant the glycosylase assay was replicated a minimum of three times. Error bars at the individual time points represent the standard deviation. The resulting data was averaged and fit to Eq. 1. The large variance in product formation for the R46Q hOGG1 variants was observed over numerous replicates of the glycosylase assay. [file 13104_2019_4111_MOESM3_ESM.pdf]

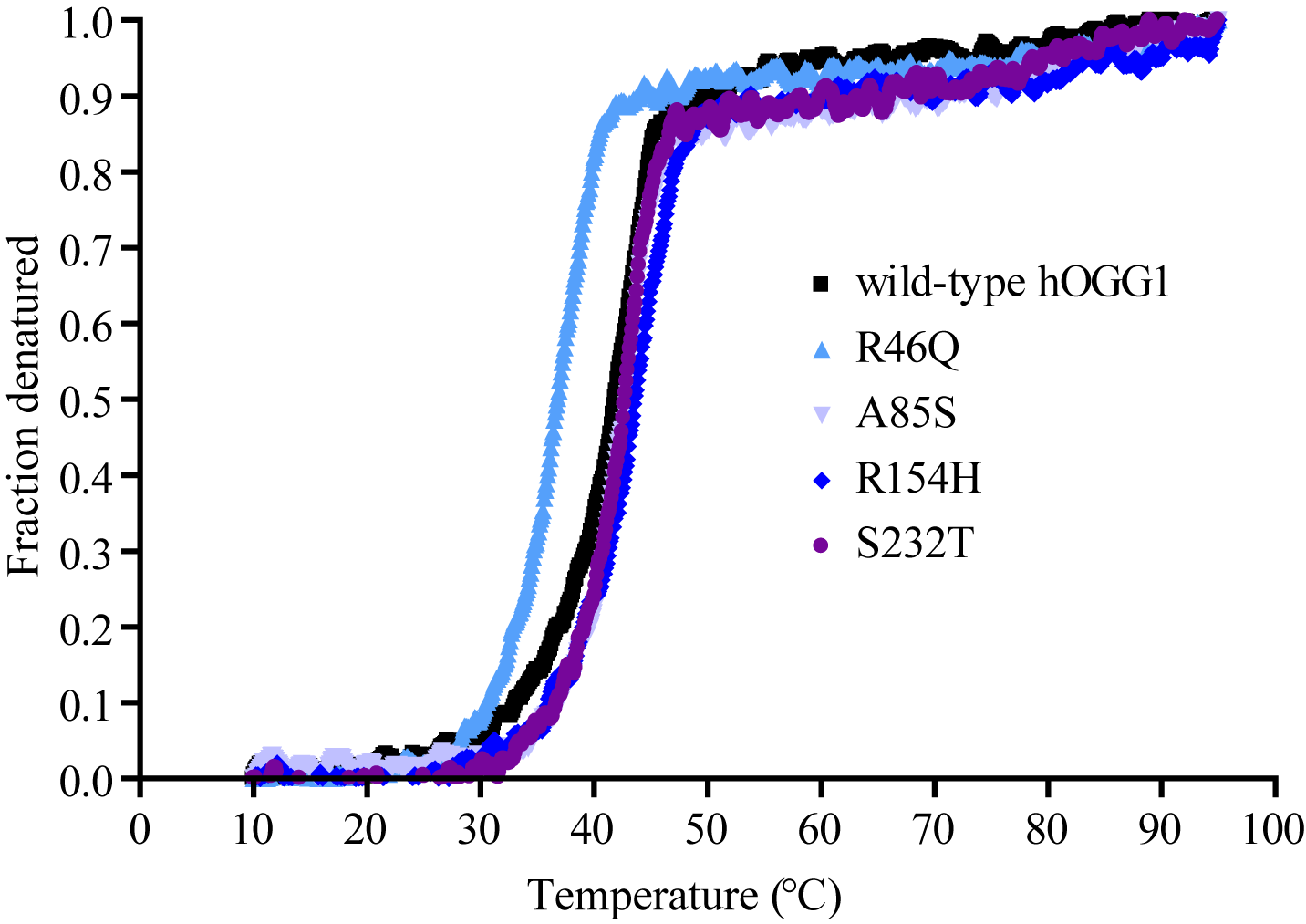

Supplement: Supplementary file 4 — Additional file 4. Thermal denaturation of hOGG1 variants. Circular dichroism (CD) spectra were recorded for each protein sample at a concentration of 0.20 mg mL−1. The molar ellipticity [θ] (degree cm2 dmol−1) at 222 nm was recorded as the temperature was increased from 10.0 to 95.0 °C at a rate of 1 °C min−1 and the resulting data was normalized to provide the fraction denatured. The average values from three replicate denaturations are plotted. [file 13104_2019_4111_MOESM4_ESM.tif]
