# Supplementary material for: Thermostability and excision activity of polymorphic forms of hOGG1
Source: BMC Res Notes. 2019 Feb 18;12:92. doi: 10.1186/s13104-019-4111-9 (PMC6379936; doi:10.1186/s13104-019-4111-9)
Supplement: Supplementary file 5 — Additional file 5. Structural analysis of the amino acid residues mutated in hOGG1 variants. Analysis based on the original published structure of hOGG1 bound to DNA [20]. [file 13104_2019_4111_MOESM5_ESM.pdf]

Structural analysis of the amino acid residues mutated in hOGG1 variants

Residue	Location	Primary contacts	Distance from oxoG[1]	Role
Arg46	Loop between αA and βB (interior core)	Glu139 (αE) His179 (βG)	14 Å	Structural
Ala85	Loop between βE and αB (surface)	Solvent	38 Å	Unknown
Arg154	αF (invades DNA helix)	Estranged cytosine	11 Å	Specificity
Ser232	Loop between αJ and αK (surface)	Solvent Glu235(αK)	22 Å	Unknown

[1]Measured from closest atom in residue to C1' of deoxyribose attached to 8oxoG base
