# Supplementary material for: Thermostability and excision activity of polymorphic forms of hOGG1
Source: BMC Res Notes. 2019 Feb 18;12:92. doi: 10.1186/s13104-019-4111-9 (PMC6379936; doi:10.1186/s13104-019-4111-9)
Supplement: Supplementary file 6 — Additional file 6. Multiple sequence alignment for OGG1 from diverse organisms. Yellow bars highlight amino acid residues that were varied in this study (R46, A85, R154, and S232). Residues that participate directly in catalysis (K249 and D268) are marked in red. The secondary structure annotation is based on the high-resolution crystal structure of K249Q hOGG1 bound to DNA [20]. The conserved HhH-GPD motif is highlighted in purple. Sequences were aligned using ClustalW2 [29]. The Genbank accession numbers for the sequences used are as follows: Homo sapiens, [GenBank:AAB61340.1]; Macaca mulatta, [GenBank:XP_001096322.1]; Bos taurus, [GenBank:NP_001073754.2]; Mus musculus, [GenBank:NP_035087.3]; Rattus norvegicus, [GenBank:NP_110497.1]; Arabidopsis thaliana, [GenBank:CAC83625.1]; Drosophila melanogaster, [GenBank:NP_572499.2]. [file 13104_2019_4111_MOESM6_ESM.pdf]

```
                                                                      bA       aA
H. SAPIENS         -----------------MPARALLPRRMGHRTLASTPALWASIPCPRSELRLDLVLPSGQ  43
M. MULATTA         -----------------MPARALLPRRMGHRTLASAPALWASIPCPRSELRLDLVLPSGQ  43
B. TAURUS          ---------------MEMSVVSLLRGSMGHRTLASFPALWASIPCPRSELRLDLVLASGQ  45
M. MUSCULUS        -----------------MLFRSWLPSSMRHRTLSSSPALWASIPCPRSELRLDLVLASGQ  43
R. NORVEGICUS      -----------------MLFSSSLSSSMRHRTLTSSPALWASIPCPRSELRLDLVLASGQ  43
A. THALIANA        MKRPRPTSQPSISSTVKPPLSPPVTPILKQKLHRTGTPKWFPLKLTHTELTLPLTFPTGQ  60
D. MELANOGASTER    ----------------MLAHNLGFHKKRLFSNMKAVLQDRGVIGLSLEECDLERTLLGGQ  44

                       bB     bC      bD        bE                     aB
H. SAPIENS         SFRWR---EQSPAHWSGVLADQVWTLTQTEEQLHCTVYRGDKSQASRPTPDELEAVRKYF  100
M. MULATTA         SFRWR---EQSPAHWSGVLADQVWTLTQTEEQLHCTVYRGDKSQPGRPTPDELEAVRKYF  100
B. TAURUS          SFRWR---EQSPAHWSGVLADQVWTLTQTEEHLYCTVYRGDKGRVGRPTLEELKAVQQYF  102
M. MUSCULUS        SFRWK---EQSPAHWSGVLADQVWTLTQTEDQLYCTVYRGDDSQVSRPTLEELETLHKYF  100
R. NORVEGICUS      SFRWR---EQSPAHWSGVLADQVWTLTQTEDQLYCTVYRGDKGQVGRPTLEELETLHKYF  100
A. THALIANA        TFRWK---KTGAIQYSGTIGPHLVSLRQRPGDDAVSYC----VHCSTSPKSAELALLDFL  113
D. MELANOGASTER    SFRWRSICDGNRTKYGGVVFNTYWVLQQEESFITYEAYG---TSSPLATKDYSSLISDYL  101

                         aC          aD                 aE                aF
H. SAPIENS         QLDVTLAQLYHHWGSVDSHFQEVAQKFQGVRLLRQDPIECLFSFICSSNNNIARITGMVE  160
M. MULATTA         QLDVTLAQLYHHWGSVDSHFQEVAQKFQGVRLLRQDPIECLFSFICSSNNNIARITGMVE  160
B. TAURUS          QLDVSLAPLYHHWSSVDPHFQEVAQKFKGVRLLQLDPIECLFSFICSSNNNIARITGMVE  162
M. MUSCULUS        QLDVSLAQLYSHWASVDSHFQRVAQKFQGVRLLRQDPTECLFSFICSSNNNIARITGMVE  160
R. NORVEGICUS      QLDVSLTQLYSHWASVDSHFQSVAQKFQGVRLLRQDPTECLFSFICSSNNNIARITGMVE  160
A. THALIANA        NAEISLAELWSDFSKKDPRFGELARHLRGARVLRQDPLECLIQFLCSSNNNIARITKMVD  173
D. MELANOGASTER    RVDFDLKVNQKDWLSKDDNFVKFLS--KPVRLLSQEPFENIFSFLCSQNNNIKRISSMIE  159

                         bF     bG        aG          aH                aI
H. SAPIENS         RLCQAFGPRLIQLDDVTYHGFPSLQALAGPEVEAH---LRKLGLGYRARYVSASARAILE  217
M. MULATTA         RLCQAFGPRLIQLDDVTYHGFPSLQALAGPEVEAH---LRKLGLGYRARYVSASARAILE  217
B. TAURUS          RLCQTFGPRLIQLDDVTYHGFPSLQALAGPEVEAQ---LRNLGLGYRARFVSASARAILE  219
M. MUSCULUS        RLCQAFGPRLIQLDDVTYHGFPNLHALAGPEAETH---LRKLGLGYRARYVRASAKAILE  217
R. NORVEGICUS      RLCQAFGPRLVQLDDVTYHGFPNLHALAGPEVETH---LRKLGLGYRARYVCASAKAILE  217
A. THALIANA        -FVSSLGLHLGDIDGFEFHQFPSLDRLS-RVSEEE---FRKAGFGYRAKYITGTVNALQA  228
D. MELANOGASTER    WFCATFGTKIGHFNGADAYTFPTINRFHDIPCEDLNAQLRAAKFGYRAKFIAQTLQEIQK  219

                         aJ         aK                aL                   aM
H. SAPIENS         EQGG-LAWLQQLRESSYEEAHKALCILPGVGTKVADCICLMALDKPQAVPVDVHMWHIAQ  276
M. MULATTA         EQGG-LPWLQQLREASYEEAHKALCTLPGVGTKVADCICLMALDKPQAVPVDVHIWQIAQ  276
B. TAURUS          ERGG-LPWLQQLRKAPYEEAHKALCTLPGVGTKVADCICLMALDKPQAVPVDVHVWQIAQ  278
M. MUSCULUS        EQGG-PAWLQQLRVAPYEEAHKALCTLPGVGAKVADCICLMALDKPQAVPVDVHVWQIAH  276
R. NORVEGICUS      EQGG-PAWLQQLRVASYEEAHKALCTLPGVGTKVADCICLMALDKPQAVPVDIHVWQIAH  276
A. THALIANA        KPGGGNEWLLSLRKVELQEAVAALCTLPGVGPKVAACIALFSLDQHSAIPVDTHVWQIAT  288
D. MELANOGASTER    KGGQ--NWFISLKSMPFEKAREELTLLPGIGYKVADCICLMSMGHLESVPVDIHIYRIAQ  277

                                   aN                       aO
H. SAPIENS         RDYSWHPTTSQAKGPSPQTNKELGNFFRSLWGPYAGWAQAVLFSADLRQSRHAQE----P  332
M. MULATTA         RDYSWHPTTSQAKGPSPQSNKELGNFFRSLWGPHAGWAQAVLFSADLHQSHRAQE----P  332
B. TAURUS          RDYSWHPTTSQAKGPSPQANKELGNFFRNLWGPYAGWAQAVLFSADLRQLQQAQE----P  334
M. MUSCULUS        RDYGWHPKTSQAKGPSPLANKELGNFFRNLWGPYAGWAQAVLFSADLRQPSLSRE----P  332
R. NORVEGICUS      RDYGWQPKTSQTKGPSPLANKELGNFFRNLWGPYAGWAQAVLFSADLRQQNLSRE----P  332
A. THALIANA        ---NYLLPDLAGAKLTPKLHGRVAEAFVSKYGEYAGWAQTLLFIAELPAQKTLLQSFSQP  345
D. MELANOGASTER    N--YYLPHLTGQKNVTKKIYEEVSKHFQKLHGKYAGWAQAILFSADLSQFQNTST-----  330

H. SAPIENS         PAKRRKGSKGPEG-------  345
M. MULATTA         PAKRRKGSKGLEV-------  345
B. TAURUS          PAKRRKRCTGPEG-------  347
M. MUSCULUS        PAKRKKGSKRPEG-------  345
R. NORVEGICUS      PAKRKKGSKKTEG-------  345
A. THALIANA        INKLDESAEVNETSCDTLKP  365
D. MELANOGASTER    VACKKKSNKKPKK-------  343
```
